# Thromboembolic adverse event study of combined estrogen-progestin preparations using Japanese Adverse Drug Event Report database

**DOI:** 10.1371/journal.pone.0182045

**Published:** 2017-07-21

**Authors:** Shiori Hasegawa, Toshinobu Matsui, Yuuki Hane, Junko Abe, Haruna Hatahira, Yumi Motooka, Sayaka Sasaoka, Akiho Fukuda, Misa Naganuma, Kouseki Hirade, Yukiko Takahashi, Yasutomi Kinosada, Mitsuhiro Nakamura

**Affiliations:** 1 Laboratory of Drug Informatics, Gifu Pharmaceutical University, Gifu, Japan; 2 Medical Database Co., Ltd., Shibuya-ku, Tokyo, Japan; 3 Kizawa Memorial Hospital, Minokamo, Gifu, Japan; 4 Critical Care and Surgical Nursing, Gifu University School of Medicine, Gifu, Japan; 5 United Graduate School of Drug Discovery and Medical Information Sciences, Gifu University, Gifu, Japan; Inselspital Universitatsspital Bern, SWITZERLAND

## Abstract

Combined estrogen-progestin preparations (CEPs) are associated with thromboembolic (TE) side effects. The aim of this study was to evaluate the incidence of TE using the Japanese Adverse Drug Event Report (JADER) database. Adverse events recorded from April 2004 to November 2014 in the JADER database were obtained from the Pharmaceuticals and Medical Devices Agency (PMDA) website (www.pmda.go.jp). We calculated the reporting odds ratios (RORs) of suspected CEPs, analyzed the time-to-onset profile, and assessed the hazard type using Weibull shape parameter (WSP). Furthermore, we used the applied association rule mining technique to discover undetected relationships such as the possible risk factors. The total number of reported cases in the JADER contained was 338,224. The RORs (95% confidential interval, CI) of drospirenone combined with ethinyl estradiol (EE, Dro-EE), norethisterone with EE (Ne-EE), levonorgestrel with EE (Lev-EE), desogestrel with EE (Des-EE), and norgestrel with EE (Nor-EE) were 56.2 (44.3–71.4), 29.1 (23.5–35.9), 42.9 (32.3–57.0), 44.7 (32.7–61.1), and 38.6 (26.3–56.7), respectively. The medians (25%–75%) of the time-to-onset of Dro-EE, Ne-EE, Lev-EE, Des-EE, and Nor-EE were 150.0 (75.3–314.0), 128.0 (27.0–279.0), 204.0 (44.0–660.0), 142.0 (41.3–344.0), and 16.5 (8.8–32.0) days, respectively. The 95% CIs of the WSP-β for Ne-EE, Lev-EE, and Nor-EE were lower and excluded 1. Association rule mining indicated that patients with anemia had a potential risk of developing a TE when using CEPs. Our results suggest that it is important to monitor patients administered CEP for TE. Careful observation is recommended, especially for those using Nor-EE, and this information may be useful for efficient therapeutic planning.

## Introduction

Combined estrogen-progestin preparations (CEPs) are one of the most commonly used birth control methods worldwide. CEPs have benefits beyond preventing an undesired pregnancy, including reduced ovarian and endometrial cancer risk, reduced dysfunctional uterine bleeding, decreased menstrual flow and menorrhagia, decreased primary dysmenorrhea, improved hirsutism and acne, and decreased risk of premenstrual syndrome/premenstrual dysphoric disorder [1].

Because CEPs are administered to healthy women over the long-term, patients should be carefully monitored for adverse events (AEs). CEPs, such as oral contraceptives (OCs), have a variety of side effects, of which thrombosis is the most frequent and important [2]. Numerous studies have demonstrated a relationship between CEPs and thromboembolism (TE), including venous thromboembolism (VTE) [1–15]. According to the American College of Obstetricians and Gynecologists, the incidence of TE increases from 1 to 5 occurrences per 10,000 women per year in non-OC users to 3 to 9 occurrences per 10,000 women per year in OC users [16]. A systematic review indicated that the risk of VTE in women of childbearing age who were non-OC users was 4 per 10,000 women per year, whereas in OC users, the risk was 7 to 10 per 10,000 women per year [4]. Appropriate treatment of TE after onset resolves the thrombus; however, in approximately 20%–50% of cases of TE, and in proximal deep vein thrombosis (DVT) to a greater extent, patients develop a post-thrombotic syndrome with lifelong problems including pain and swelling of the leg [17,18]. Rare thrombi cause pulmonary embolism, and 1 in 100 cases results in death [13].

Few studies have examined the association between CEP use and arterial thromboembolism (ATE), such as myocardial infarction and ischemic stroke [2,10,19–23]. Although ATE is less frequent than VTE, the consequences of ATE are often more serious [23]. The World Health Organization (WHO) has reported that the use of CEPs increased the risk of myocardial infarction by approximately 5-fold and the risk of ischemic stroke by approximately 3-fold [2,19,20].

Because VTE and ATE are rare AEs associated with CEP use, the implementation phase of epidemiologic research is difficult. The Pharmaceuticals and Medical Devices Agency (PMDA) in Japan has released the Japanese Adverse Drug Event Report (JADER) database, which is a large spontaneous reporting system (SRS) and reflects the realities of clinical practice in Japan [24]. Therefore, JADER has been used for pharmacovigilance assessments for rare AEs using the reporting odds ratio (ROR) [24–27].

Several studies have indicated that the risk of developing CEP-induced VTE is greatest during the first year of use [2,4,6,7,9,10,12]. However, detailed onset profiles of CEP-induced VTE are not clear. The analysis of time-to-onset data has been proposed as a new method of detecting signals for AEs in SRSs [24,27,28]. In this study, we applied the index of ROR to TE and evaluated time-to-onset profiles of TE for CEPs in the real world.

Furthermore, association rule mining has been proposed as a new analytical approach for identifying undetected clinical factor combinations, such as possible risk factors, between variables in huge databases [29–31]. This is the first application of association rule mining for the detection of association rules between CEPs and TE.

## Materials and methods

AEs recorded from April 2004 to November 2014 in the JADER database were obtained from the PMDA website (www.pmda.go.jp). The JADER database consists of 4 tables: patient demographic information, such as sex, age, and reporting year (demo); drug information, such as non-proprietary name of the prescribed drug, route, and start and end date of administration (drug); adverse events, such as type, outcome, and onset date (reac); and primary disease (hist). We constructed a relational database that integrated the 4 data tables using FileMaker Pro 12 software (FileMaker, Inc., Santa Clara, CA, USA). The “drug” file included the role codes assigned to each drug: suspected, concomitant, and interacting drugs (higiyaku, heiyouyaku, and sougosayou in Japanese, respectively). The suspected drug records were extracted and analyzed in this study.

Five CEPs (drospirenone combined with ethinyl estradiol (EE, Dro-EE), norethisterone with EE (Ne-EE), levonorgestrel with EE (Lev-EE), desogestrel with EE (Des-EE), and norgestrel with EE (Nor-EE)) were assessed. Since EE is not a constituent of Menoaid (ASKA Pharmaceutical Co., Ltd., Tokyo, Japan) and Wellnara (Bayer Yakuhin, Ltd., Osaka, Japan), they were excluded in the analysis. The number of reported cases of E·P·Hormone depot (ASKA Pharmaceutical Co., Ltd., Tokyo, Japan), Lutes depot (Mochida Pharmaceutical Co., Ltd., Tokyo, Japan), Lutedion (ASKA Pharmaceutical Co., Ltd., Tokyo, Japan), and Sophia (ASKA Pharmaceutical Co., Ltd., Tokyo, Japan) were low and, therefore, they were not assessed in the analysis.

AEs in the JADER database are coded according to the terminology preferred by the Medical Dictionary for Regulatory Activities/Japanese version 17.1 (MedDRA/J) (www.pmrj.jp/jmo/php/indexj.php). The standardized MedDRA Queries (SMQ) index consists of groupings of MedDRA terms, ordinarily at the preferred term (PT) level, that relate to a defined medical condition or area of interest [32]. We used the SMQ for embolic and thrombotic events, arterial (SMQ code: 20000082), embolic and thrombotic events, vessel type unspecified and mixed arterial and venous (SMQ code: 20000083), and embolic and thrombotic events, venous (SMQ code: 20000084; Table 1).

**Table 1:** Preferred terms of thromboembolism associated with combined estrogen-progestin preparations in MedDRA ^a)^.

Embolic and thrombotic events, arterial		Embolic and thrombotic events, vessel type unspecified and mixed arterial and venous		Embolic and thrombotic events, venous	
(SMQ ^b)^ code: 20000082)		(SMQ ^b)^ code: 20000083)		(SMQ ^b)^ code: 20000084)	
CODE	Preferred Term	CODE	Preferred Term	CODE	Preferred Term
10074337	Acute aortic syndrome	10075178	Adrenal thrombosis	10003880	Axillary vein thrombosis
10000891	Acute myocardial infarction	10060956	Angiogram abnormal	10006537	Budd-Chiari syndrome
10001902	Amaurosis	10052906	Angiogram cerebral abnormal	10052698	Catheterisation venous
10001903	Amaurosis fugax	10057517	Angiogram peripheral abnormal	10007830	Cavernous sinus thrombosis
10002475	Angioplasty	10058562	Arteriovenous fistula occlusion	10053377	Central venous catheterisation
10057617	Aortic bypass	10003192	Arteriovenous fistula thrombosis	10008138	Cerebral venous thrombosis
10002897	Aortic embolus	10048632	Atrial thrombosis	10053681	Compression stockings application
10061651	Aortic surgery	10071043	Basal ganglia stroke	10051055	Deep vein thrombosis
10002910	Aortic thrombosis	10049824	Bone infarction	10066881	Deep vein thrombosis postoperative
10057794	Aortogram abnormal	10074422	Brain stem embolism	10014522	Embolism venous
10071026	Arterectomy	10006147	Brain stem infarction	10058991	Hepatic vein occlusion
10003140	Arterectomy with graft replacement	10068644	Brain stem stroke	10019713	Hepatic vein thrombosis
10056418	Arterial bypass operation	10062573	Brain stem thrombosis	10051031	Homans' sign positive
10061655	Arterial graft	10053994	Cardiac ventricular thrombosis	10058992	Iliac vein occlusion
10062599	Arterial occlusive disease	10067167	Cerebellar embolism	10070911	Inferior vena cava syndrome
10061657	Arterial stent insertion	10008034	Cerebellar infarction	10058987	Inferior vena caval occlusion
10052949	Arterial therapeutic procedure	10008118	Cerebral infarction	10061251	Intracranial venous sinus thrombosis
10003178	Arterial thrombosis	10008119	Cerebral infarction foetal	10023237	Jugular vein thrombosis
10061659	Arteriogram abnormal	10008120	Cerebral ischaemia	10075428	Mahler sign
10003195	Arteriogram carotid abnormal	10070671	Cerebral septic infarct	10069727	May-Thurner syndrome
10063025	Atherectomy	10008132	Cerebral thrombosis	10027402	Mesenteric vein thrombosis
10069020	Basal ganglia infarction	10052173	Cerebrospinal thrombotic tamponade	10027403	Mesenteric venous occlusion
10048963	Basilar artery occlusion	10008190	Cerebrovascular accident	10029925	Obstetrical pulmonary embolism
10063093	Basilar artery thrombosis	10049165	Cerebrovascular accident prophylaxis	10073708	Obstructive shock
10005184	Blindness transient	10008196	Cerebrovascular disorder	10074349	Ophthalmic vein thrombosis
10069694	Brachiocephalic artery occlusion	10051902	Cerebrovascular operation	10072059	Ovarian vein thrombosis
10067744	Capsular warning syndrome	10057403	Choroidal infarction	10050216	Paget-Schroetter syndrome
10071260	Carotid angioplasty	10069729	Collateral circulation	10034272	Pelvic venous thrombosis
10007684	Carotid arterial embolus	10059025	Coronary bypass thrombosis	10034324	Penile vein thrombosis
10053003	Carotid artery bypass	10074896	Device embolisation	10048874	Phlebectomy
10048964	Carotid artery occlusion	10064685	Device occlusion	10073979	Portal vein cavernous transformation
10066102	Carotid artery stent insertion	10013033	Diplegia	10058989	Portal vein occlusion
10007688	Carotid artery thrombosis	10013048	Directional Doppler flow tests abnormal	10036206	Portal vein thrombosis
10007692	Carotid endarterectomy	10013442	Disseminated intravascular coagulation	10063909	Post procedural pulmonary embolism
10053633	Cerebellar artery occlusion	10013443	Disseminated intravascular coagulation in newborn	10048591	Post thrombotic syndrome
10008023	Cerebellar artery thrombosis	10060839	Embolic cerebral infarction	10050902	Postoperative thrombosis
10008088	Cerebral artery embolism	10065680	Embolic pneumonia	10036300	Postpartum venous thrombosis
10008089	Cerebral artery occlusion	10014498	Embolic stroke	10037377	Pulmonary embolism
10008092	Cerebral artery thrombosis	10061169	Embolism	10037410	Pulmonary infarction
10065384	Cerebral hypoperfusion	10053601	Foetal cerebrovascular disorder	10037421	Pulmonary microemboli
10058842	Cerebrovascular insufficiency	10051269	Graft thrombosis	10037437	Pulmonary thrombosis
10061751	Cerebrovascular stenosis	10019005	Haemorrhagic cerebral infarction	10068690	Pulmonary vein occlusion
10069696	Coeliac artery occlusion	10019013	Haemorrhagic infarction	10037458	Pulmonary veno-occlusive disease
10050329	Coronary angioplasty	10019016	Haemorrhagic stroke	10037459	Pulmonary venous thrombosis
10052086	Coronary arterial stent insertion	10055677	Haemorrhagic transformation stroke	10038547	Renal vein embolism
10011077	Coronary artery bypass	10019023	Haemorrhoids thrombosed	10056293	Renal vein occlusion
10011084	Coronary artery embolism	10019465	Hemiparesis	10038548	Renal vein thrombosis
10011086	Coronary artery occlusion	10019468	Hemiplegia	10038907	Retinal vein occlusion
10053261	Coronary artery reocclusion	10062506	Heparin-induced thrombocytopenia	10038908	Retinal vein thrombosis
10011091	Coronary artery thrombosis	10019680	Hepatic infarction	10068479	SI QIII TIII pattern
10011101	Coronary endarterectomy	10074494	Hepatic vascular thrombosis	10068122	Splenic vein occlusion
10049887	Coronary revascularization	10063868	Implant site thrombosis	10041659	Splenic vein thrombosis
10075162	Coronary vascular graft occlusion	10061216	Infarction	10049446	Subclavian vein thrombosis
10058729	Embolia cutis medicamentosa	10065489	Infusion site thrombosis	10042567	Superior sagittal sinus thrombosis
10014513	Embolism arterial	10022104	Injection site thrombosis	10058988	Superior vena cava occlusion
10014648	Endarterectomy	10070754	Inner ear infarction	10042569	Superior vena cava syndrome
10068365	Femoral artery embolism	10073625	Instillation site thrombosis	10043570	Thrombophlebitis
10052019	Femoral artery occlusion	10022657	Intestinal infarction	10043581	Thrombophlebitis migrans
10019635	Hepatic artery embolism	10066087	Intracardiac mass	10043586	Thrombophlebitis neonatal
10051991	Hepatic artery occlusion	10048620	Intracardiac thrombus	10043595	Thrombophlebitis superficial
10019636	Hepatic artery thrombosis	10027401	Mesenteric vascular insufficiency	10043605	Thrombosed varicose vein
10063518	Hypothenar hammer syndrome	10074583	Mesenteric vascular occlusion	10067270	Thrombosis corpora cavernosa
10021338	Iliac artery embolism	10073734	Microembolism	10044457	Transverse sinus thrombosis
10064601	Iliac artery occlusion	10027925	Monoparesis	10067740	Vascular graft
10052989	Intra-aortic balloon placement	10027926	Monoplegia	10047193	Vena cava embolism
10056382	Intraoperative cerebral artery occlusion	10030936	Optic nerve infarction	10048932	Vena cava filter insertion
10060840	Ischaemic cerebral infarction	10068239	Pancreatic infarction	10074397	Vena cava filter removal
10061256	Ischaemic stroke	10066059	Paradoxical embolism	10047195	Vena cava thrombosis
10051078	Lacunar infarction	10033885	Paraparesis	10047209	Venogram abnormal
10024242	Leriche syndrome	10033892	Paraplegia	10062173	Venoocclusive disease
10027394	Mesenteric arterial occlusion	10033985	Paresis	10047216	Venoocclusive liver disease
10065560	Mesenteric arteriosclerosis	10053351	Peripheral revascularization	10058990	Venous occlusion
10027395	Mesenteric artery embolism	10035092	Pituitary infarction	10062175	Venous operation
10027396	Mesenteric artery stenosis	10064620	Placental infarction	10068605	Venous recanalisation
10071261	Mesenteric artery stent insertion	10059829	Pneumatic compression therapy	10052964	Venous repair
10027397	Mesenteric artery thrombosis	10036204	Portal shunt	10063389	Venous stent insertion
10028596	Myocardial infarction	10066591	Post procedural stroke	10047249	Venous thrombosis
10028602	Myocardial necrosis	10068628	Prosthetic vessel implantation	10067030	Venous thrombosis in pregnancy
10033697	Papillary muscle infarction	10049680	Quadriparesis	10061408	Venous thrombosis limb
10068035	Penile artery occlusion	10037714	Quadriplegia	10064602	Venous thrombosis neonatal
10065608	Percutaneous coronary intervention	10038470	Renal infarct		
10062585	Peripheral arterial occlusive disease	10072226	Renal vascular thrombosis		
10069379	Peripheral arterial reocclusion	10051742	Retinal infarction		
10057518	Peripheral artery angioplasty	10062108	Retinal vascular thrombosis		
10072561	Peripheral artery bypass	10040621	Shunt occlusion		
10072562	Peripheral artery stent insertion	10059054	Shunt thrombosis		
10072564	Peripheral artery thrombosis	10058571	Spinal cord infarction		
10061340	Peripheral embolism	10041648	Splenic infarction		
10072560	Peripheral endarterectomy	10074601	Splenic thrombosis		
10071642	Popliteal artery entrapment syndrome	10074515	Stoma site thrombosis		
10066592	Post procedural myocardial infarction	10058408	Surgical vascular shunt		
10058144	Postinfarction angina	10043337	Testicular infarction		
10036511	Precerebral artery occlusion	10064961	Thalamic infarction		
10074717	Precerebral artery thrombosis	10043530	Thrombectomy		
10063731	Pulmonary artery therapeutic procedure	10043540	Thromboangiitis obliterans		
10037340	Pulmonary artery thrombosis	10043568	Thrombolysis		
10072893	Pulmonary endarterectomy	10043607	Thrombosis		
10057493	Renal artery angioplasty	10062546	Thrombosis in device		
10048988	Renal artery occlusion	10043626	Thrombosis mesenteric vessel		
10038380	Renal artery thrombosis	10043634	Thrombosis prophylaxis		
10063544	Renal embolism	10067347	Thrombotic cerebral infarction		
10038826	Retinal artery embolism	10043647	Thrombotic stroke		
10038827	Retinal artery occlusion	10043742	Thyroid infarction		
10038831	Retinal artery thrombosis	10045168	Tumour embolism		
10049768	Silent myocardial infarction	10068067	Tumour thrombosis		
10049440	Spinal artery embolism	10061604	Ultrasonic angiogram abnormal		
10071316	Spinal artery thrombosis	10045413	Ultrasound Doppler abnormal		
10074600	Splenic artery thrombosis	10071652	Umbilical cord thrombosis		
10068677	Splenic embolism	10069922	Vascular graft thrombosis		
10066286	Stress cardiomyopathy	10049071	Vascular operation		
10059613	Stroke in evolution	10063382	Vascular stent insertion		
10042332	Subclavian artery embolism	10058794	Vasodilation procedure		
10069695	Subclavian artery occlusion	10070649	Vessel puncture site thrombosis		
10042334	Subclavian artery thrombosis	10066856	Visual midline shift syndrome		
10054156	Superior mesenteric artery syndrome				
10064958	Thromboembolectomy				
10043645	Thrombotic microangiopathy				
10043648	Thrombotic thrombocytopenic purpura				
10044390	Transient ischaemic attack				
10062363	Truncus coeliacus thrombosis				
10048965	Vertebral artery occlusion				
10057777	Vertebral artery thrombosis				
10047532	Visual acuity reduced transiently				

^a)^ Medical Dictionary for Regulatory Activities

^b)^ Standardized MedDRA Queries

The mosaic plot of the two-way frequency table was constructed with the age-category (X) and primary disease (Y). A mosaic plot is divided into rectangles so that the vertical length of each rectangle is proportional to the proportion of the Y variable at each level of the X variable.

**Fig 1:**
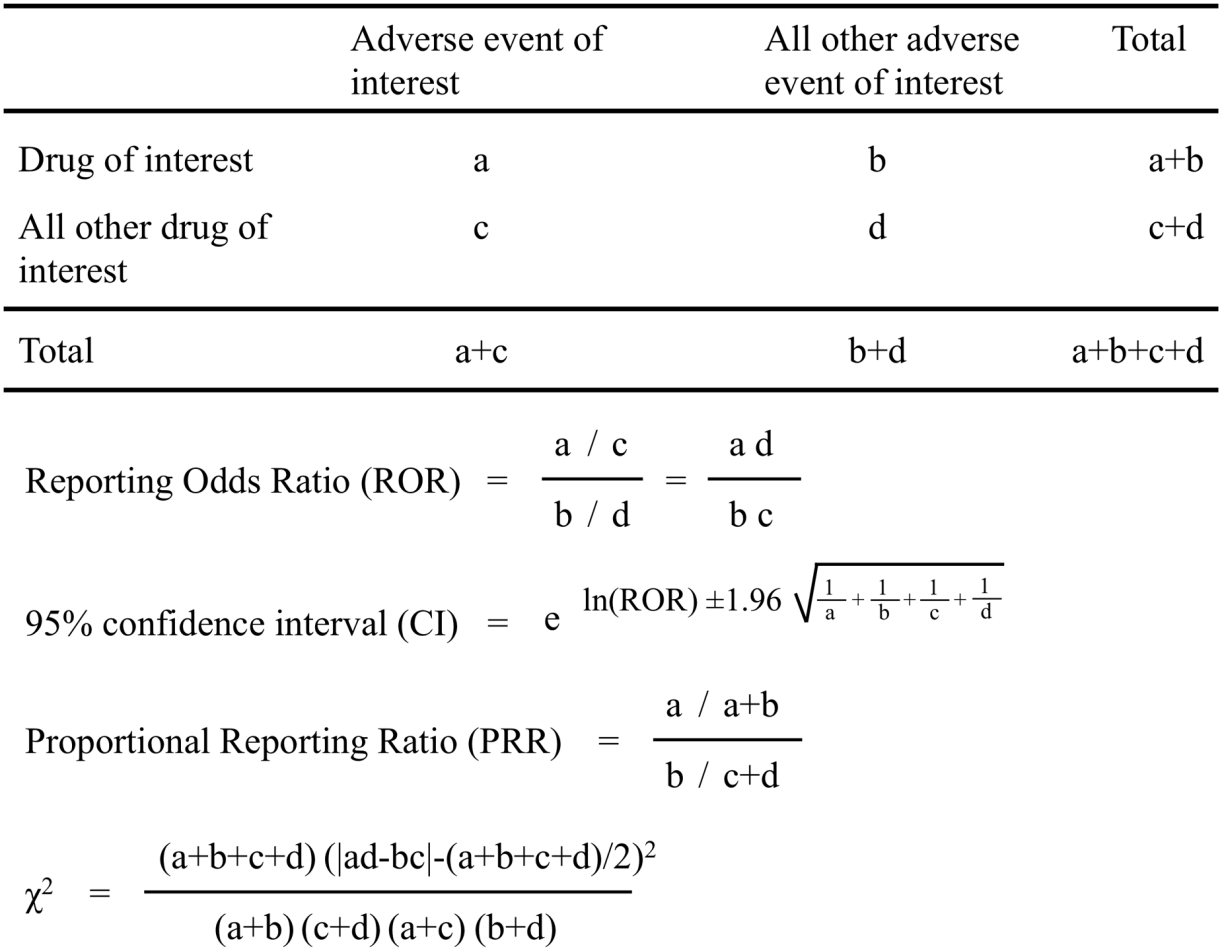
Two by two table used for the calculation of reporting odds ratios and proportional reporting ratio.

We assessed the association between CEPs and TE using the ROR, which is an established parameter for pharmacovigilance research. The ROR is the ratio of the odds of reporting an adverse event versus all other events associated with the drug of interest compared with the reporting odds for all other drugs present in the database [33]. We calculated the ROR using a two-by-two contingency table by defining the rows using CEPs and all other drugs and the columns using TE and all other adverse events (Fig 1). RORs are expressed as point estimates with 95% confidence intervals (CI). The detection of a signal was dependent on the signal indices exceeding a predefined threshold. Safety signals are considered significant when the ROR estimates and the lower limits of the corresponding 95% CI exceed 1. At least 2 cases are required to define a signal [33,34].

Proportional reporting ratios (PRRs) are measures of disproportionality used for detecting signals in SRS databases [35]. PRRs are calculated from the same 2 × 2 tables and the ROR is identical to the calculation of relative risk (RR) from a cohort study, i.e., [a / (a + c)] / [b / (b + d)]. If the drug and adverse event are independent, the expected value of the PRR is 1. The minimum criteria for signal detection are as follows: 3 or more cases, PRR of at least 2, and Chi-square of at least 4.

Time-to-onset duration of the data from the JADER database was calculated from the time of the patient’s first prescription to the occurrence of the AE. The median duration, quartiles, and Weibull shape parameters (WSPs) were used to evaluate the dates from administration to development of TE [27,36–38]. The WSP test is used for the statistical analysis of time-to-onset data and can describe the non-constant rate of the incidence of AE reactions [24,39]. The scale parameter α of the Weibull distribution determines the scale of the distribution function. A larger scale value stretches the distribution. A smaller scale value shrinks the data distribution. The shape parameter β of the Weibull distribution indicates the hazard without a reference population. When β is equal to 1, the hazard is estimated to be constant over time. When β is greater than 1 and the 95% CI of β excludes 1, the hazard is considered to increase over time. When β is smaller than 1 and the 95% CI of β excludes 1, the hazard is considered to decrease over time [39]. The data analyses were performed using JMP 11.2 (SAS Institute Inc., Cary, NC, USA).

### Association rule mining

The association rule mining approach attempts to search the frequent items in databases and discover interesting relationships between variables. Given a set of transactions ***T*** (each transaction is a set of items), an association rule can be expressed as X -> Y, where X and Y are mutually exclusive sets of items [40]. The rule’s statistical significance and strength are measured by the *support* and *confidence*, respectively. *Support* is defined as the percentage of transactions in the data that contain all items in both the antecedent (left-hand-side of rule: lhs) and the consequent of the rule (right-hand-side of rule: rhs) [40]. The support indicates how frequently the rule occurs in the transaction. The formula for calculating *support* is as follows:

Support =P(X ∩ Y)={X ∩ Y}/{D}

D is the total number of the transaction. *Confidence* corresponds to the conditional probability P (Y|X). A rule with high *confidence* is important because it provides an accurate prediction of the association of the items in the rule. The formula for calculating *confidence* is as follows:

Confidence =P(X ∩ Y)/P(X)

*Lift* represents the ratio of probability. For a given rule, X and Y occur together to the multiple of the two individual probabilities for X and Y; that is,

Lift =P(X ∩ Y)/P(X) P(Y)

Since P(Y) appears in the denominator of the *lift* measure, the *lift* can be expressed as the confidence divided by P(Y). The *lift* can be evaluated as follows: *lift* = 1, if X and Y are independent; *lift* > 1, if X and Y are positively correlated; *lift* < 1, if X and Y are negatively correlated. We performed these analyses using the apriori function of the *arules* library in the *arules* package of R version 3.3.3 software [41].

## Results

The JADER database contained 338,224 reports from April 2004 to November 2014. The number of reports including TE was 14,593. The RORs (95% CI) of agents with Dro-EE, Ne-EE, Lev-EE, Des-EE, and Nor-EE were 56.2 (44.3–71.4), 29.1 (23.5–35.9), 42.9 (32.3–57.0), 44.7 (32.7–61.1), and 38.6 (26.3–56.7), respectively (Table 2). The PRRs (95% CI) of Dro-EE, Ne-EE, Lev-EE, Des-EE, and Nor-EE were 16.8 (13.2–21.3), 13.2 (10.7–16.4), 15.4 (11.6–20.4), 15.6 (11.4–21.3), and 14.8 (10.0–21.7), respectively (Table 2).

**Table 2:** Number of reports, proportional reporting ratio and reporting odds ratio of thromboembolism ^a)^.

Drug	Age (years old)	Case	Total	Non-case	Reporting ratio of thromboembolism (%)	PRR (95% CI)	χ2	ROR (95% CI)
Total		14593	338224					
All CEPs ^b)^		744	1163	419	64.0	15.6 (13.8−17.6)	10046.1	41.4 (36.7−46.8)
	10−19	14	30	16	46.7	10.8 (5.3−22.2)	120.3	19.4 (9.5−39.8)
	20−29	113	198	85	57.1	13.3 (10.1−17.7)	1322.9	29.7 (22.4−39.4)
	30−39	243	355	112	68.5	16.1 (12.9−20.2)	3525.3	48.9 (39.1−61.2)
	40−49	298	400	102	74.5	17.6 (14.1−22.1)	4761.4	66.1 (52.8−82.8)
	50−59	35	52	17	67.3	15.6 (8.8−27.9)	484.7	45.8 (25.6−81.7)
Drospirenone-EE ^c)^		237	332	95	71.4	16.8 (13.2−21.3)	3604.9	56.2 (44.3−71.4)
	10−19	7	12	5	58.3	13.5 (4.3−42.6)	72.2	31.1 (9.9−97.9)
	20−29	37	52	15	71.2	16.5 (9.1−30.1)	546.7	54.8 (30.1−99.9)
	30−39	79	98	19	80.6	18.8 (11.4−31.0)	1363.8	92.7 (56.2−153.0)
	40−49	86	101	15	85.1	19.8 (11.5−34.4)	1579.5	127.9 (73.9−221.4)
	50−59	12	14	2	85.7	19.9 (4.4−88.8)	205.4	133.2 (29.8−595.1)
Norethisterone-EE ^c)^		198	351	153	56.4	13.2 (10.7−16.4)	2297.2	29.1 (23.5−35.9)
	10−19	2	2	0	100.0	−*	−*	−^†^
	20−29	20	54	34	37.0	8.6 (4.9−14.9)	132.2	13.1 (7.5−22.7)
	30−39	54	99	45	54.5	12.7 (8.5−18.8)	593.1	26.7 (18.0−39.7)
	40−49	108	155	47	69.7	16.3 (11.5−22.9)	1588.9	51.3 (36.4−72.3)
	50−59	6	12	6	50.0	11.6 (3.7−35.9)	50.1	22.2 (7.2−68.8)
Levonorgestrel-EE ^c)^		140	213	73	65.7	15.4 (11.6−20.4)	1932.2	42.9 (32.3−57.0)
	10−19	1	4	3	25.0	−*	−*	−**
	20−29	19	32	13	59.4	13.8 (6.8−27.9)	221.9	32.5 (16.0−65.7)
	30−39	54	77	23	70.1	16.3 (10.0−26.6)	792.2	52.3 (32.1−85.2)
	40−49	56	76	20	73.7	17.1 (10.3−28.6)	869.3	62.3 (37.4−103.9)
	50−59	7	11	4	63.6	14.8 (4.3−50.4)	79.9	38.8 (11.4−132.7)
Desogestrel-EE ^c)^		118	177	59	66.7	15.6 (11.4−21.3)	1652.6	44.7 (32.7−61.1)
	10−19	3	7	4	42.9	9.9 (2.2−44.4)	16.7	16.6 (3.7−74.3)
	20−29	25	43	18	58.1	13.5 (7.4−24.7)	288.9	30.9 (16.8−56.6)
	30−39	43	54	11	79.6	18.5 (9.5−35.9)	723.9	87.0 (44.8−168.6)
	40−49	34	43	9	79.1	18.4 (8.8−38.3)	564.2	84.0 (40.3−175.1)
	50−59	2	3	1	66.7	−*	−*	44.4 (4.0−489.3)
Norgestrel-EE ^c)^		71	112	41	63.4	14.8 (10.0−21.7)	932.9	38.6 (26.3−56.7)
	10−19	1	5	4	20.0	−*	−*	−**
	20−29	12	19	7	63.2	14.6 (5.8−37.2)	145.4	38.1 (15.0−96.7)
	30−39	25	39	14	64.1	14.9 (7.7−28.6)	323.4	39.7 (20.6−76.3)
	40−49	22	33	11	66.7	15.5 (7.5−31.9)	295.9	44.4 (21.5−91.6)
	50−59	8	12	4	66.7	15.5 (4.7−51.3)	98.4	44.4 (13.4−147.4)

^a)^ SMQ code (20000082, 20000083, and 20000084)

^b)^ CEP: Combined Estrogen-progestin Preparations.

^c)^ EE: Ethinyl Estradiol.

* Number of cases < 3.

** Number of cases < 2.

^†^Non-case was not reported.

**Fig 2:**
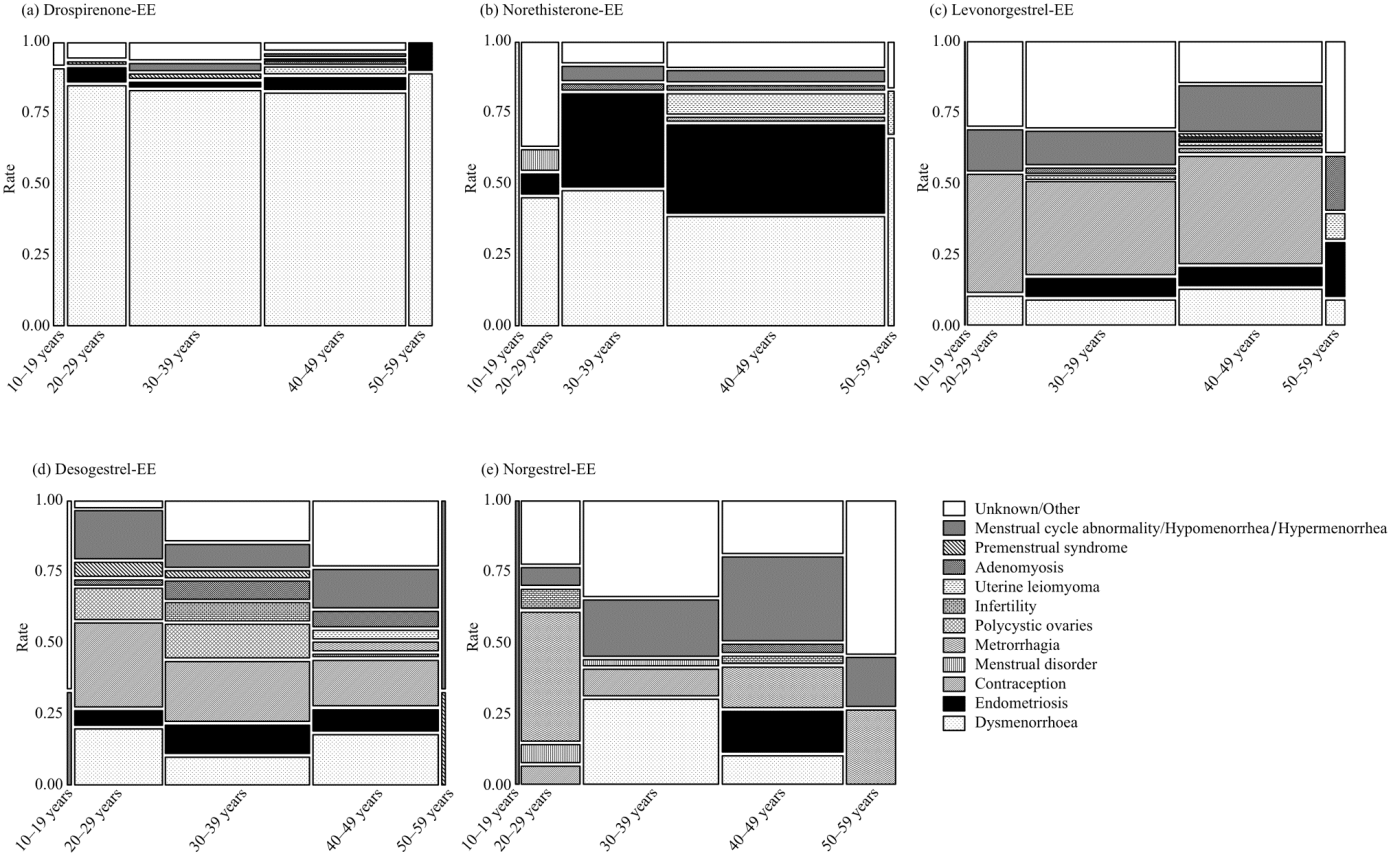
Mosaic plot of thromboembolism by combined estrogen-progestin preparations.

In the mosaic plot, Dro-EE and Nor-EE were primarily administered to patients with dysmenorrhea and endometriosis, respectively (Fig 2). The ROR and 95% CI of patients stratified by age in the 10–19, 20–29, 30–39, 40–49, and 50–59 -year-old groups were 19.4 (9.5–39.8), 29.7 (22.4–39.4), 48.9 (39.1–61.2), 66.1 (52.8–82.8), and 45.8 (25.6–81.7), respectively (Table 2).

The analysis of time-to-onset profiles revealed that the median values (25%–75%) of thrombosis caused by agents containing Dro-EE, Ne-EE, Lev-EE, Des-EE, and Nor-EE were 150.0 (75.3–314.0), 128.0 (27.0–279.0), 204.0 (44.0–660.0), 142.0 (41.3–344.0), and 16.5 (8.8–32.0) days, respectively (Table 3). The WSP β (95% CI) for Dro-EE, Ne-EE, Lev-EE, Des-EE, and Nor-EE were 1.12 (1.01–1.23), 0.81 (0.72–0.91), 0.81 (0.68–0.96), 0.92 (0.78–1.07), and 0.62 (0.50–0.75), respectively (Table 3).

**Table 3:** Quartiles and parameter of Weibull distribution and failure pattern for combined estrogen-progestin preparations.

Drugs	Case reports (n)	Median (lower−upper quartile)(day)	Scale parameter, α (95% CI)	Shape parameter, β (95% CI)
*Total*	670	135.0 (40.0−305.3)	202.0 (184.0−221.5)	0.86 (0.81−0.91)
Drospirenone-EE	240	150.0 (75.3−314.0)	223.8 (198.2−252.2)	1.12 (1.01−1.23)
Norethisterone-EE	185	128.0 (27.0−279.0)	186.3 (153.8−224.6)	0.81 (0.72−0.91)
Levonorgestrel-EE	95	204.0 (44.0−660.0)	281.4 (214.7−365.5)	0.81 (0.68−0.96)
Desogestrel-EE	100	142.0 (41.3−344.0)	225.9 (179.1−282.8)	0.92 (0.78−1.07)
Norgestrel-EE	50	16.5 (8.8−32.0)	38.3 (23.3−61.8)	0.62 (0.50−0.75)
*Subgroup for contraception*				
Subtotal	67	244.0 (104.0−730.0)	333.8 (258.5−427.0)	1.03 (0.83−1.25)
Drospirenone-EE	−	−	−	−
Norethisterone-EE	−	−	−	−
Levonorgestrel-EE	42	245.0 (172.5−730.0)	408.8 (303.1−545.2)	1.14 (0.86−1.46)
Desogestrel-EE	25	183.0 (31.5−370.5)	238.1 (150.7−366.6)	0.99 (0.70−1.35)
Norgestrel-EE	−	−	−	−
*Subgroup for dysmenorrhea*				
Subtotal	324	137.0 (58.3−292.3)	208.0 (184.3−234.1)	0.96 (0.88−1.05)
Drospirenone-EE	204	150.0 (69.3−345.8)	223.5 (195.1−255.3)	1.09 (0.97−1.21)
Norethisterone-EE	88	126.0 (27.8−287.3)	200.2 (151.2−262.6)	0.81 (0.68−0.95)
Levonorgestrel-EE	13	88.0 (14.0−250.0)	166.1 (91.2−291.3)	1.27 (0.71−2.05)
Desogestrel-EE	12	135.5 (79.0−262.0)	217.4 (126.0−363.5)	1.28 (0.79−1.87)
Norgestrel-EE	7	26.0 (16.0−197.0)	87.5 (26.0−273.4)	0.85 (0.42−1.47)
*Subgroup for endomeriosis*				
Subtotal	88	123.0 (40.3−329.8)	219.3 (158.3−300.7)	0.70 (0.59−0.82)
Drospirenone-EE	13	134.0 (76.5−314.0)	184.2 (107.3−305.4)	1.25 (0.75−1.91)
Norethisterone-EE	57	106.0 (23.5−253.0)	167.9 (116.0−239.4)	0.78 (0.63−0.95)
Levonorgestrel-EE	6	240.0 (6.0−669.3)	502.1 (192.9−1275.4)	1.55 (0.54−3.36)
Desogestrel-EE	9	679.0 (125.0−730.0)	563.4 (353.9−876.8)	1.93 (0.94−3.48)
Norgestrel-EE	3	19.0 (19.0−20.0)	−	−

**Fig 3:**
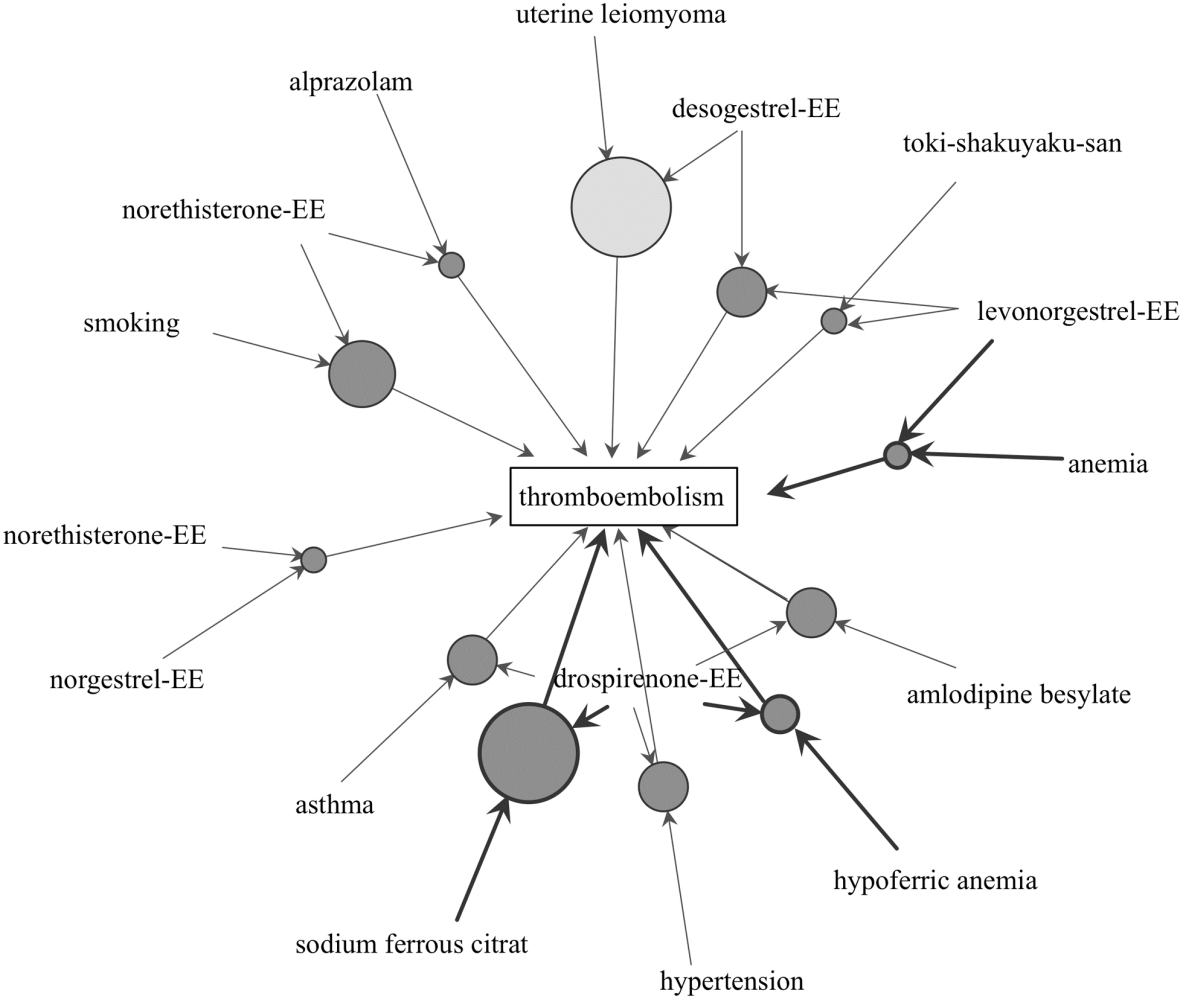
Association rules of thromboembolism by combined estrogen-progestin preparations. The plot represents items and rules as vertices connected with directed edges. Relation parameters are typically added to the plot as labels on the edges or by varying the color or width of the arrows indicating the edges.

The association rule mining technique was applied to TE (as consequent) using demographic data such as age category and patient history. The apriori algorithm extracts frequent combinations from a large database to efficiently find sets of adverse events that occur more frequently than the minimum *support* threshold (defined as 0.00001 in this study). This generates sets of adverse drug reactions with the minimum *confidence* threshold (defined as 0.9 in this study). Furthermore, the maximum size of mined frequent item sets (*maxlen*: a parameter in the *arules* package) was restricted to 3. The result of the mining algorithm was a set of 12 rules (Table 4). The *support*, *confidence*, and *lift* of each association rule are summarized in Table 4; the association rules up to the twelfth position in descending order of the *support* are shown in Table 4. {Des-EE, uterine leiomyoma} -> {TE} demonstrated a high *support* value (Table 4, id [1]). The association rules of {sodium ferrous citrate, Dro-EE} -> {TE}, {Dro-EE, hypoferric anemia} -> {TE}, and {Lev-EE, anemia} -> {TE} with high scores for *lift* and *support* were demonstrated (Table 4 (id [2], [8], [11], Fig 3). Additionally, the association rules of the combination of {smoking, Nor-EE} were high (Table 4, id [3]).

**Table 4:** Association parameters of rules (sorted by support).

id	lhs ^a)^		rhs ^b)^	support	confidence	Lift
[1]	{Desogestrel-EE, uterine leiomyoma}	→	{thromboembolism}	0.000030	0.91	21.07
[2]	{sodium ferrous citrate, Drospirenone-EE}	→	{thromboembolism}	0.000030	1.00	23.18
[3]	{smoking, Norethisterone-EE}	→	{thromboembolism}	0.000021	1.00	23.18
[4]	{Desogestrel-EE, Levonorgestrel-EE}	→	{thromboembolism}	0.000018	1.00	23.18
[5]	{Drospirenone-EE, asthma}	→	{thromboembolism}	0.000018	1.00	23.18
[6]	{Amlodipine besylate, Drospirenone-EE}	→	{thromboembolism}	0.000018	1.00	23.18
[7]	{Drospirenone-EE, hypertension}	→	{thromboembolism}	0.000018	1.00	23.18
[8]	{Drospirenone-EE, hypoferric anemia}	→	{thromboembolism}	0.000015	1.00	23.18
[9]	{Norethisterone-EE, Norgestrel-EE}	→	{thromboembolism}	0.000012	1.00	23.18
[10]	{Levonorgestrel-EE, toki-shakuyaku-san}	→	{thromboembolism}	0.000012	1.00	23.18
[11]	{Levonorgestrel-EE, anemia}	→	{thromboembolism}	0.000012	1.00	23.18
[12]	{alprazolam, Norethisterone-EE}	→	{thromboembolism}	0.000012	1.00	23.18

^a)^ left-hand-sides of rule (antecedents)

^b)^ right-hand-side (consequents)

## Discussion

The RORs and PRRs suggested that all CEPs were associated with an increased risk of TE. Several studies demonstrated that the increase in VTE risk after administration of Dro-EE or Des-EE was greater than that after administration of Lev-EE [6,8,11,15,42]. The risk of VTE might be associated with the type of progestin, the amount of estrogen, or the pharmacological activity of estrogen [6,7]. In contrast, Odlind et al. suggested that those associations might be subject to bias [43,44]. Whereas some studies indicated that Des-EE reduced the risk of ATE compared to other CEPs, other studies did not [2,23]. Lidegaard et al. found the risk of ATE decreased with lower doses of estrogen [23]. We did not observe significant differences in the RORs among Dro-EE, Nor-EE, Lev-EE, and Des-EE. We do not have a conclusive explanation for the differences in TE risk between the various progestins in low-dose CEPs.

The median time to TE onset induced by Nor-EE, which contained the highest amount of EE (50 μg), was the shortest time to onset among the CEPs (16.5 days). EE enhances the effects of the procoagulation factors 2, 7, 9, 10, 12, 13, and fibrinogen, while reducing natural anticoagulant protein S and antithrombin, and acts as a procoagulant [2,45]. The effects of EE were reported to be dose-dependent [2]. With an estrogen dose of 30 μg as the reference category, the thrombotic risk was 0.8 (95% CI 0.5 to 1.2) for an estrogen dose of 20 μg and 1.9 (1.1 to 3.4) for a dose of 50 μg [11]. In contrast, progestin has no effect on coagulation factor levels [2]. One plausible reason for the “short” median time to TE onset induced by Nor-EE might be the high amount of EE in Nor-EE in our study. However, the mechanism of development of thrombosis is poorly understood. It may be due to the differential effects on sex hormone binding globulin, anticoagulant protein S resistance in early OC use, or the unmasking of an underlying inherited coagulation disorder [4]. CEPs have several metabolic effects on lipid, carbohydrate, and hemostatic parameters [3]. To reveal the mechanism of the short time to onset of TE by Nor-EE, further pharmacological study is necessary.

The WSP β of Ne-EE, Lev-EE, and Nor-EE was less than 1, which indicated an early failure type, and indicated that TE caused by these CEPs might decrease over time. It was reported that the risk of VTE decreased with prolonged administration [46–48] and recovered to the level of non-users of CEPs within 3 months after discontinuation [15].

In our study, the median occurrence of TE for all CEPs was within 3 months; however, several instances of VTE were observed after 3 months. The risks of VTE were reported to be observed within 4 months following CEP administration [15]. These results corresponded with those of previous studies and confirmed the necessity of long-term observation after the administration of these drugs.

In the association rule mining, because the *lift* values of two combined items, CEPs and anemia-related items, including iron pill administration, were high, patients with anemia had a potential risk of TE when using CEPs. Recently, an association between anemia and cerebral venous thrombosis was reported [49]. Therefore, anemia patients should be monitored carefully. The *lift* values of the two combined items, {smoking, Nor-EE}, were also high enough to suggest an association. This information demonstrated that smoking while taking CEPs may increase the risk of TE.

Association rule mining is one of the most important tasks in data mining and various effective algorithms have been proposed. Several groups have conducted the performance evaluation of the association rule mining algorithms, such as apriori, Frequent Pattern (FP)-Growth, and Eclat, by execution time or those with higher confidence, lift, and conviction values. Apriori is a level-wise, breadth-first algorithm that counts transactions, generates candidates, and discovers frequent itemsets by the exploitation of user-specified support and confidence measures. In a large quantity of itemsets, the algorithm requires more space and time; consequently, the complexity of the algorithm increases [50]. The FP-Growth algorithm was proposed as an alternative to the apriori-based approach by Han [51,52]. The basic concept of the FP-Growth algorithm consists of the construction of an FP-tree for all the transactions. FP-Growth encodes the data set by using a compact data structure called an FP-tree, which can save considerable amounts of memory in transaction storage [52,53]. The Eclat algorithm uses equivalence classes, depth-first search, and set intersection instead of counting. Eclat is a depth-first search-based algorithm that uses a vertical database layout [54]. It also solves the frequent itemset problem. However, the performance by each algorithm differs owing to various parameters, such as the size of itemset and the structure of database. We consider that the relative merits of the algorithms have not yet been settled.

An apriori algorithm is designed to efficiently identify association rules in large databases and is the most classical algorithm for mining frequent item sets [55]. This algorithm has recently been used for the analysis of AEs in the JADER and US Food and Drug Administration (FDA) Adverse Event Reporting System (FAERS) and confirmed its usefulness for pharmacovigilance [29–31]. Therefore, we used an apriori algorithm.

The numerous known risk factors for TE in women are as follows: advanced age [5,6,11], high body mass index [14,47,56–60], smoking [20,61–65], breast cancer, migraine, hypertension, and medical history of a cardiovascular event [1,66]. CEP use should be discouraged among women older than 35 years who smoke because they have an increased risk of arterial vascular disease when using CEP [10]. CEP users should have their blood pressure routinely monitored and smoking cessation should be encouraged in older women. Clinicians should monitor for any symptoms suggestive of stroke, myocardial infarction, or venous thrombosis and discontinue the agent immediately if any symptoms occur during the first 3 months of CEP use. From our results, Nor-EE users should be closely monitored for the first 2 to 3 weeks. Regarding the prescribing of CEPs, clinicians should consider a woman's risk factors for TE. The choice of an appropriate CEP should be made by considering the need to minimize the risk of TE, patient preference, and available alternatives.

Like the JADER database, the FAERS database is an SRS and is the largest and best-known AEs database worldwide. Therefore, the FDA uses it for pharmacovigilance activities, such as looking for new safety concerns that might be related to a drug. The FAERS database files are publicly available on the FDA web site (open.fda.gov/data/faers/) [33]. FAERS includes information about the country where the AEs occurred. From our preliminary analysis of the FAERS database from April 2004 to November 2014, the total number of reported cases in the FAERS database was 6,165,659 and the number of reports from the US and Japan was 3,652,497 (59.2%) and 275,268 (4.5%), respectively (detailed data not shown). The number of reported AEs in the JADER (338,224 in this study) was greater than that in the FAERS (275,268 from Japan). Nomura et al. reported that there are differences in the reported number of AEs between JADER and FAERS, but the reports that were common between the FAERS and JADER were uncertain [67]. SRS databases mostly depend on the compliance of pharmaceutical companies to report according to regulatory requirements. Each company has its own operational rules for AE reports, which makes it impossible for researchers to validate the contents of SRS databases [67]. Regional differences in drug prescriptions or genetic backgrounds may be related to AEs. However, we did not analyze this issue further.

The JADER database does not contain detailed background information regarding patients’ body mass index, smoking, or accurate medical history, such as migraine and cardiovascular disease. Furthermore, SRS has several limitations, including under-reporting, over-reporting, missing data, bias, confounding factors, and lack of a control population as a reference group [34]. Further epidemiological studies for confirmation might be required.

Several pharmacovigilance indexes have been developed to detect drug-associated AEs, including the ROR used by the PMDA and the Netherlands Pharmacovigilance Centre (Lareb), the PRR used by the Medicines and Healthcare Products Regulatory Agency in the United Kingdom (UK), the information component (IC) used by WHO, and the empirical Bayes geometric mean (EBGM) used by the FDA. The multi-item gamma poisson shrinker (MGPS) method is a disproportionality method that utilizes an empirical Bayesian model to detect the magnitude of drug-event associations in drug safety databases [68,69]. MGPS calculates adjusted reporting ratios for pairs of drug event combinations. The adjusted reporting ratio values are termed the EBGM. Although many studies regarding the performance, accuracy, and reliability of different data mining algorithms are in progress, there is no recognized *gold standard* methodology. We did not analyze using the EBGM, but this might be a future consideration.

The ROR is defined as the ratio of the odds of reporting of one specific event versus all other events for a given drug compared to the reporting odds for all other drugs present in the database. Basically, the higher the value, the stronger the disproportion appears to be. The ROR indicates an increased risk of AE reporting and not a risk of AE occurrence. Therefore, the ROR does not allow risk quantification, but only offers a rough indication of signal strength and is only relevant to the hypothesis [24,33,34]. The ROR is a clear and easily applicable technique that allows for the control of confounding factors through logistic regression analysis [27,70–72]. An additional advantage of using the ROR is that non-selective underreporting of a drug or AE has no influence on the value of the ROR compared with the population of patients experiencing an AE [73]. Therefore, we selected first the ROR as a pharmacovigilance index in this study.

ROR and PRR are both measures of disproportionality used to detect signals in SRS databases. In our study, the tendencies of the results from the RORs and the PRRs for signal detection were similar. Evans et al. suggested that the PRR might be much less error prone than the ROR [35]. In contrast, Rothman et al. proposed that SRS should be treated as a data source for a case-control study, thereby excluding from the control series those events that may be related to drug exposure. Therefore, the ROR may offer an advantage over PRR by estimating the relative risk [74]. However, this apparent superiority has been called into question [75]. Van Puijenbroek et al. concluded that, in practice, there is no important difference between the ROR and PRR measures for pharmacovigilance [34]. A judgment on the validity and utility of these measures should be based on comparison of their sensitivity, specificity, and predictive values in signal detection from a real dataset.

The aforementioned limitations inherent to the SRS should be recognized in the interpretation of the results from the JADER database. We stress that our results do not provide any justification for the restriction of CEP use because the benefits and tolerability of CEPs have been accepted worldwide.

## Conclusion

This study was the first to evaluate the correlation between CEP and TE using an SRS analysis strategy. Despite the limitations inherent to SRS, we showed the potential risk of TE during CEP use in a real-life setting. The present analysis demonstrated that the incidence of TE with Nor-EE use should be closely monitored for a short onset (within 3 weeks). Patients with anemia who are using CEPs might be advised to adhere to an appropriate care plan. We recommend the close monitoring of patients, and those who experience any symptoms suggestive of TE should be advised to discontinue administration.
